# Supplementary material for: Tracking Epidermal Cortisol and Oxytocin in Managed Bottlenose Dolphins as Potential Non-Invasive Physiological Welfare Indicators
Source: Animals (Basel). 2025 Sep 8;15(17):2628. doi: 10.3390/ani15172628 (PMC12427215; doi:10.3390/ani15172628)
Supplement: Supplementary file 1 [file animals-15-02628-s001.zip › animals-3806232-supplementary.pdf]

Supplementary information:

Supplementary Table S1. Kruskal-Wallis and Wilcoxon rank-sum post-hoc analysis of epidermal cortisol concentrations in common bottlenose dolphins (*Tursiops truncatus*) by Season. Bonferroni correction was applied, and significant p-values ($p < 0.05$) are in bold.

Time lag	Kruskal-Wallis			Post-hoc Wilcoxon rank-sum (*p* values)					
	**Statistic**	**df**	***p*-value**	**Fall–Spring**	**Fall–Summer**	**Fall–Winter**	**Spring–Summer**	**Spring–Winter**	**Summer–Winter**
**20-26**	13.42	3	**0.004**	**< 0.001**	0.078	**0.038**	0.176	0.202	0.771
**27-33**	15.09	3	**0.002**	**< 0.001**	0.322	0.093	**0.026**	0.093	0.448
**34-40**	15.78	3	**0.001**	**0.001**	0.294	0.071	**0.015**	0.103	0.294
**41-47**	13.59	3	**0.004**	**0.008**	0.774	0.097	**0.008**	0.334	0.097
**48-54**	12.45	3	**0.006**	**0.039**	0.680	0.439	**0.009**	0.172	0.172
**55-61**	13.44	3	**0.004**	**0.036**	0.456	0.456	**0.005**	0.132	0.132
**62-68**	11.33	3	**0.010**	0.192	0.192	0.418	**0.017**	0.573	**0.028**
**69-75**	10.25	3	**0.017**	0.218	0.218	0.233	**0.025**	0.848	**0.025**

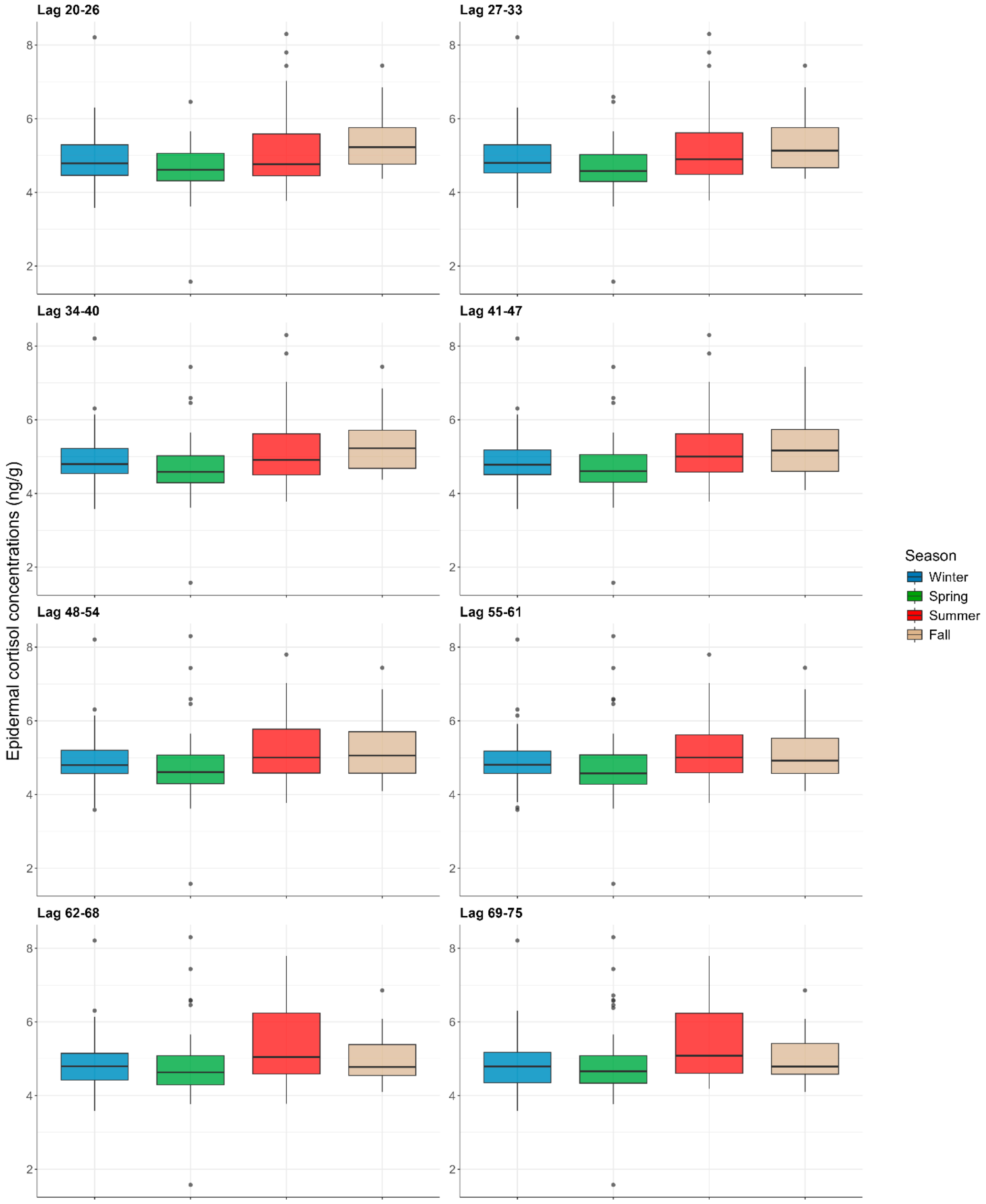

Supplementary Figure S2. Epidermal cortisol concentrations in bottlenose dolphins (*Tursiops truncatus*) across seasons (Winter, Spring, Summer and Fall), shown for different time lags between season-assigned weeks and epidermis sampling. Each panel represents one of the eight time lag intervals considered: 20–26, 27–33, 34–40, 41–47, 48–54, 55–61, 62–68, and 69–75 days before hormone sampling. Horizontal lines represent the lower quartile, median, and upper quartile values, while whiskers indicate the range.

Supplementary Table S3. Kruskal-Wallis and Wilcoxon rank-sum post-hoc analysis of epidermal cortisol concentrations in common bottlenose dolphins (*Tursiops truncatus*) by Visitor-based seasonality. Bonferroni correction was applied, and significant p-values ($p < 0.05$) are in bold.

Time lag	Kruskal-Wallis			Post-hoc Wilcoxon rank-sum (*p* values)		
	**Statistic**	**df**	***p*-value**	**Off-peak season – Peak season**	**Off-peak season – Closure period**	**Peak season – Closure period**
**20-26**	9.836	2	**0.007**	0.520	**0.010**	**0.010**
**27-33**	10.701	2	**0.005**	0.376	**0.005**	**0.005**
**34-40**	7.015	2	**0.030**	0.220	**0.050**	**0.038**
**41-47**	7.640	2	**0.022**	0.137	0.052	**0.030**
**48-54**	11.923	2	**0.003**	**0.020**	**0.031**	**0.006**
**55-61**	9.147	2	**0.010**	**0.039**	0.055	**0.030**
**62-68**	11.574	2	**0.003**	**0.017**	**0.029**	**0.017**
**69-75**	8.591	2	**0.014**	0.084	**0.037**	**0.033**

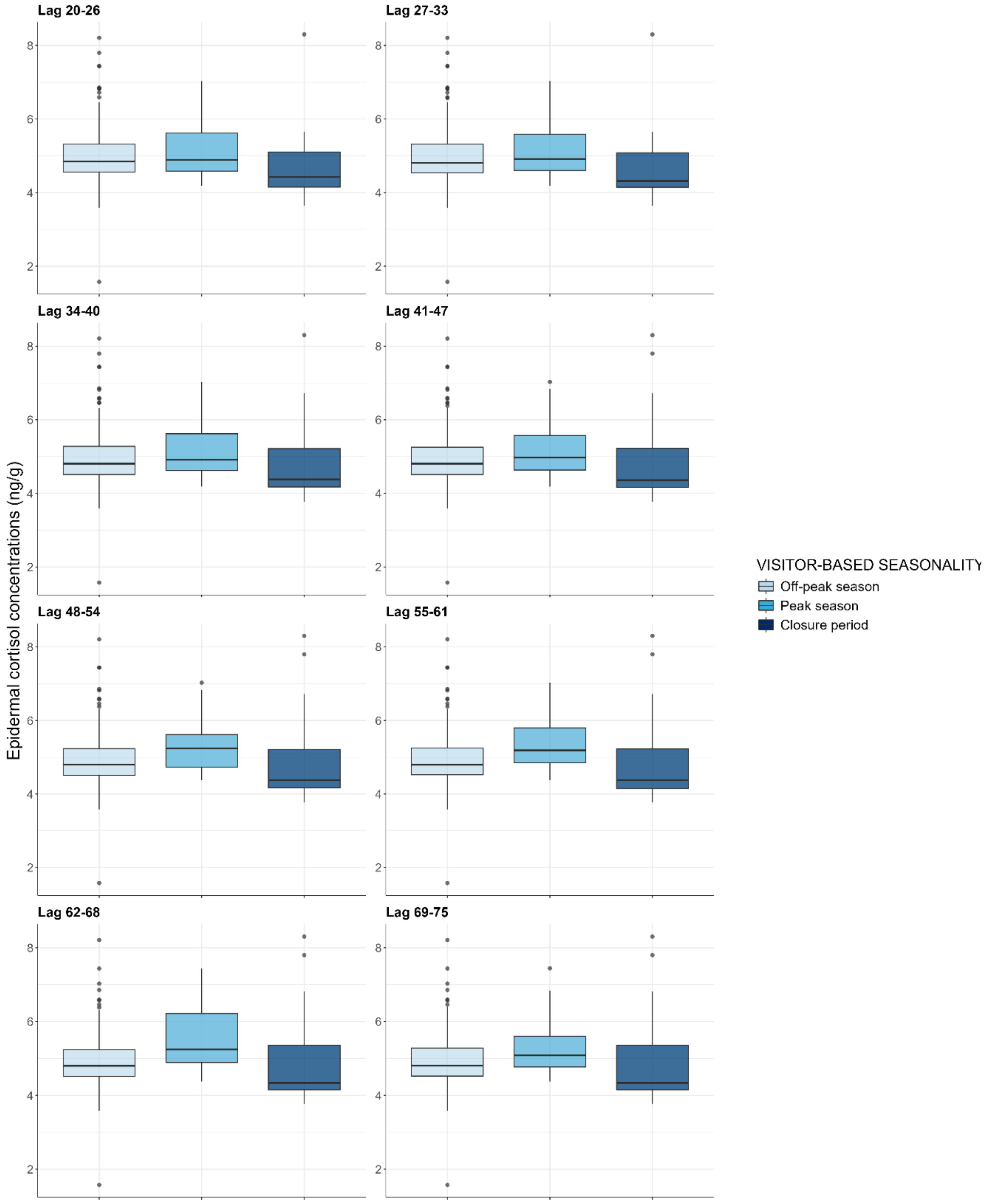

Supplementary Figure S4. Epidermal cortisol concentrations in bottlenose dolphins (*Tursiops truncatus*) across Visitor-based seasonality categories (Peak season, Off-peak season, and Closure period), shown for different time lags between categories-assigned weeks and epidermis sampling. Weeks were classified into three visitor-based seasonal categories: *Peak season*, *Off-peak season*, and *Closure period*. Each panel represents one of the eight-time lag intervals considered: 20–26, 27–33, 34–40, 41–47, 48–54, 55–61, 62–68, and 69–75 days before hormone sampling. Horizontal lines represent the lower quartile, median, and upper quartile values, while whiskers indicate the range.

Supplementary Table S5. Kruskal-Wallis and Wilcoxon rank-sum post-hoc analysis of epidermal oxytocin concentrations in common bottlenose dolphins (*Tursiops truncatus*) by Season. Bonferroni correction was applied, and significant *p*-values ($p < 0.05$) are in bold.

**Time lag**	**Kruskal-Wallis**			**Post-hoc Wilcoxon rank-sum (*p* values)**					
	**Statistic**	**df**	***p*-value**	**Fall–Spring**	**Fall–Summer**	**Fall–Winter**	**Spring–Summer**	**Spring–Winter**	**Summer–Winter**
**20-26**	14.42	3	**0.002**	**< 0.001**	**0.014**	**0.014**	0.345	0.263	0.912
**27-33**	12.99	3	**0.005**	**0.003**	**0.056**	**0.056**	0.159	0.159	0.664
**34-40**	13.85	3	**0.003**	**0.004**	**0.036**	**0.027**	0.111	0.229	0.471
**41-47**	13.67	3	**0.003**	**0.005**	**0.046**	**0.01**	0.124	0.522	0.32
**48-54**	12.40	3	**0.006**	**0.005**	0.116	0.083	0.095	0.211	0.539
**55-61**	13.11	3	**0.004**	**0.005**	0.226	0.096	0.053	0.174	0.490
**62-68**	10.11	3	**0.018**	**0.031**	0.476	0.117	0.058	0.328	0.328
**69-75**	10.89	3	**0.012**	**0.02**	0.360	**0.02**	0.12	0.86	0.12

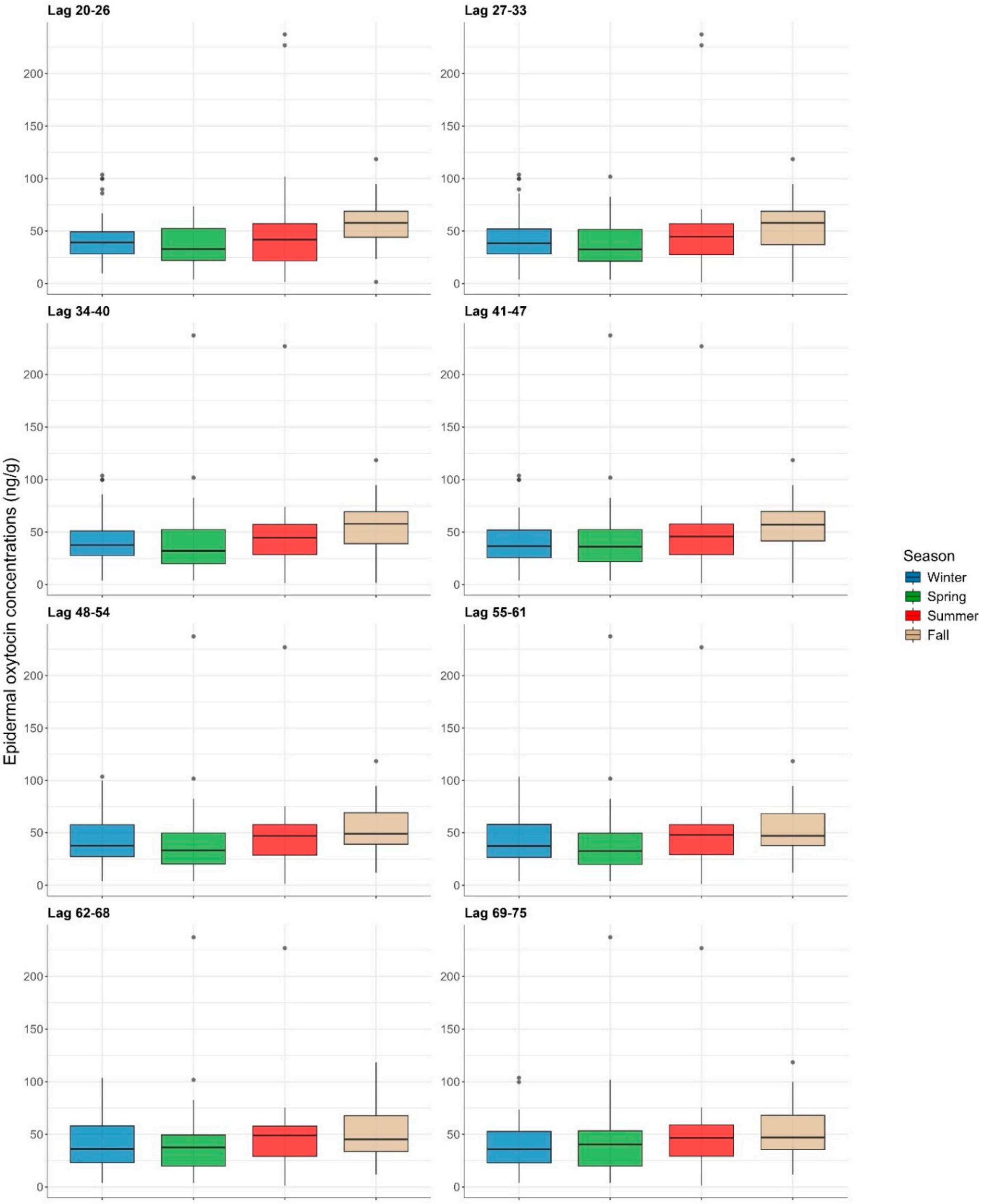

Supplementary figure S6. Epidermal oxytocin concentrations in bottlenose dolphins (*Tursiops truncatus*) across seasons (Winter, Spring, Summer and Fall), shown for different time lags between season-assigned weeks and epidermis sampling. Each panel represents one of the eight time lag intervals considered: 20–26, 27–33, 34–40, 41–47, 48–54, 55–61, 62–68, and 69–75 days before hormone sampling. Horizontal lines represent the lower quartile, median, and upper quartile values, while whiskers indicate the range.

Supplementary Table S7. Kruskal-Wallis and Wilcoxon rank-sum post-hoc analysis of epidermal oxytocin in common bottlenose dolphins (*Tursiops truncatus*) by Visitor-based seasonality. Bonferroni correction was applied, and significant *p*-values ($p < 0.05$) are in bold.

**Time lag**	**Kruskal-Wallis**			**Post-hoc Wilcoxon rank-sum (*p* values)**		
	**Statistic**	**df**	***p*-value**	**Off-peak season – Peak season**	**Off-peak season – Closure period**	**Peak season – Closure period**
**20-26**	4.532	2	0.104	0.310	0.150	0.110
**27-33**	5.667	2	0.059	0.220	0.110	0.060
**34-40**	4.824	2	0.090	0.198	0.198	0.072
**41-47**	5.824	2	0.054	0.211	0.115	**0.042**
**48-54**	7.704	2	**0.021**	0.147	0.052	**0.024**
**55-61**	11.208	2	**0.004**	0.064	**0.016**	**0.006**
**62-68**	15.010	2	**0.001**	**0.030**	**0.004**	**0.002**
**69-75**	12.671	2	**0.002**	0.137	**0.004**	**0.004**

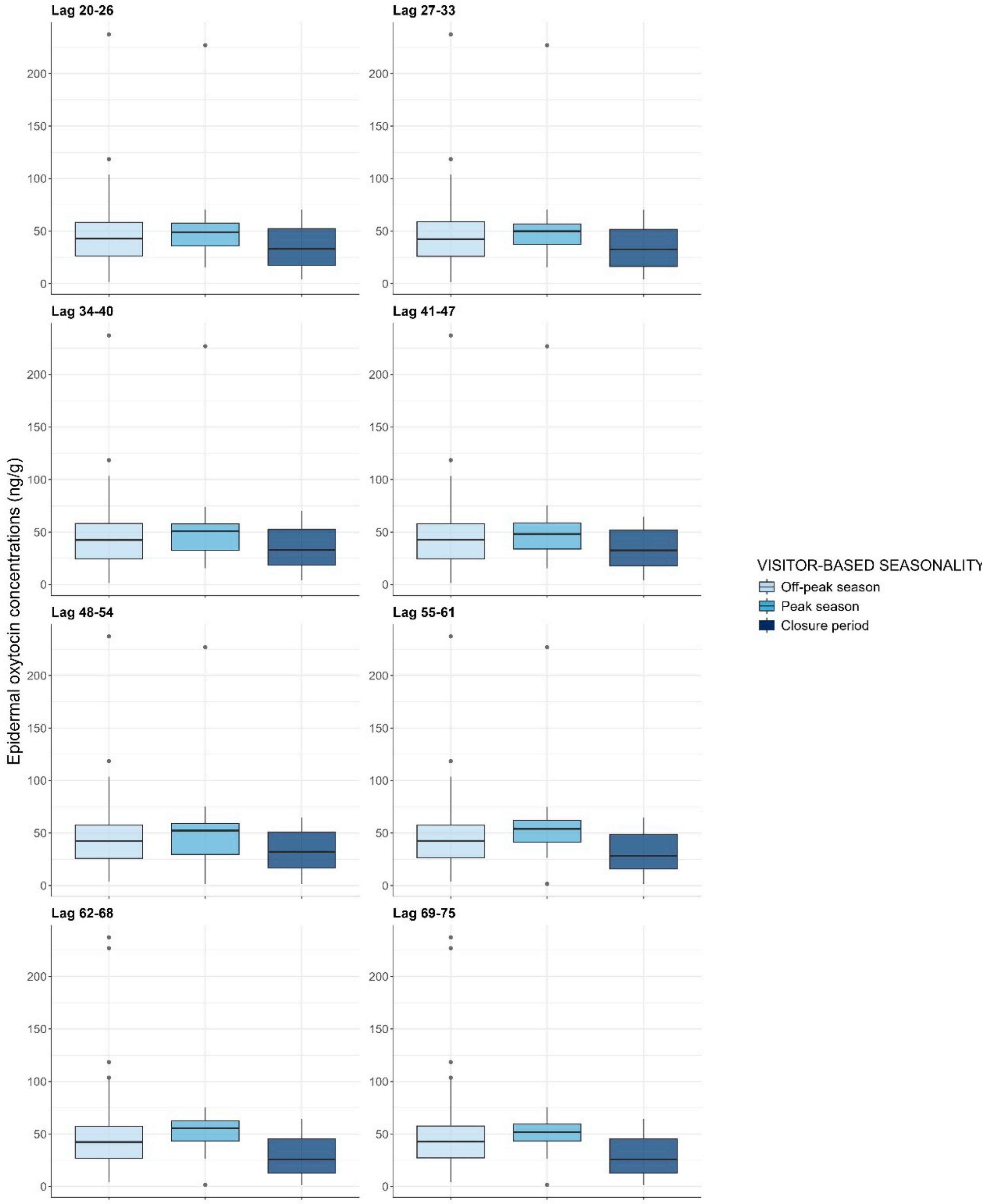

Supplementary figure S8. Epidermal oxytocin concentrations in bottlenose dolphins (*Tursiops truncatus*) across Visitor-based seasonality categories (Peak season, Off-peak season, and Closure period), shown for different time lags between categories-assigned weeks and epidermis sampling. Weeks were classified into three visitor-based seasonal categories: *Peak season*, *Off-peak season*, and *Closure period*. Each panel represents one of the eight-time lag intervals considered: 20–26, 27–33, 34–40, 41–47, 48–54, 55–61, 62–68, and 69–75 days before hormone sampling. Horizontal lines represent the lower quartile, median, and upper quartile values, while whiskers indicate the range.
